# The Montreal Cognitive Assessment at the Framingham Heart Study: A Re‐Examination of the Norms

**DOI:** 10.1002/brb3.71487

**Published:** 2026-05-11

**Authors:** Emma Muller, Calvin Guan, Katherine A. Gifford, Preeti Sunderaraman, Sherral Devine, Yulin Liu, Phillip H. Hwang, Ashita S. Gurnani

**Affiliations:** ^1^ The Framingham Heart Study Boston University School of Medicine Boston Massachusetts USA; ^2^ Department of Anatomy and Neurobiology Boston University School of Medicine Boston Massachusetts USA; ^3^ Department of Mathematics and Statistics Boston University Boston Massachusetts USA; ^4^ Department of Neurology Boston University Chobanian & Avedisian School of Medicine Boston Massachusetts USA; ^5^ Department of Epidemiology Boston University School of Public Health Boston Massachusetts USA

**Keywords:** cognitive assessment, diagnostic accuracy, mild cognitive impairment, Montreal Cognitive Assessment, normative data

## Abstract

**Objectives:**

There is a lack of consensus regarding what constitutes cognitively normal performance on the Montreal Cognitive Assessment (MoCA) based on demographic characteristics. Further, research regarding normative data on the MoCA for middle‐aged individuals is relatively limited. The current study sought to provide age‐ and education‐corrected normative data for the MoCA in a large epidemiological cohort of cognitively healthy middle‐aged and older adults with characteristics similar to the original validation sample of the MoCA.

**Methods:**

Participants were from Generation 3 and Omni 2 cohorts of the Framingham Heart Study (*n* = 2637; 91.43% non‐Hispanic White) who were determined to be cognitively unimpaired at the time of MoCA assessment (Mean age = 53.56 years, age range = 32–83 years, 63.71% ≥ college‐educated). Normative data were generated by age in 10‐year intervals and education (≤ high school, some college, or ≥ college degree). Analysis of variance was used to examine the relationship between MoCA performance, age, and education.

**Results:**

The average MoCA score across all participants was close to the revised MCI cutoff of 23 (M = 24.69, SD = 3.03). The average MoCA score for individuals over the age of 60 was below the recently suggested MCI cutoff score of 23 points. Similarly, individuals above the age of 70 scored below the revised cutoff score of 23 points, irrespective of level of education. Further, performance of participants below the age of 40 who were college educated was similar to the frequently used original MCI cutoff score of 26 (M = 26.28, SD = 2.41).

**Conclusions:**

Results are consistent with previous literature suggesting that the original MoCA cutoff score of 26 may result in a high rate of false positives. Findings indicate that the recently suggested MCI cutoff score of 23 on the MoCA may also be artificially high. Using inappropriate normative data for the MoCA can impact diagnostic accuracy as well as misclassification in research settings. These findings highlight the need for the use of demographically appropriate, population‐based normative data for the MoCA in clinical and research settings.

## Graphical abstract

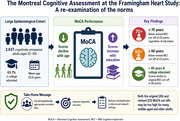

## 1 Introduction

The ineffectiveness of therapeutics in treating dementia has been attributed in part to the administration of drugs late in the neuropathological process when neuronal damage is irreversible, thereby highlighting the importance of early diagnosis of mild cognitive impairment (MCI) when interventions may be most beneficial (J. L. Cummings et al. 2014; J. Cummings et al. 2023; Folch et al. 2018; Gauthier et al. 2013; Fišar and Hroudová et al. 2024). Currently, early detection of MCI is largely dependent on the use of traditional cognitive screening instruments such as the Montreal Cognitive Assessment (MoCA), which is frequently used in clinical trials, practice, and research settings (Carton et al. 2025; Cersonsky et al. 2022; Dautzenberg et al. 2020; Dautzenberg et al. 2021; Nasreddine et al. 2005). Developed in 1996, the MoCA evaluates several cognitive domains, has a maximum score of 30 points, and takes 10–15 min to administer, with an original suggested cutoff of < 26 points for MCI (Nasreddine et al. 2005). However, despite its widespread use, the MoCA has been criticized for its cultural bias and potentially high cutoff scores (Ratcliffe et al. 2023; Stimmel et al. 2024).

Specifically, several studies have shown that the original recommended cutoff score of 26 on the MoCA results in a high rate of false positives, particularly in those with older age or low education (Ratcliffe et al. 2023, 2024; Carson et al. 2017; Davis et al. 2015; Stimmel et al. 2024). The recommended one‐point correction for education on the MoCA has also been shown to insufficiently compensate for educational differences and may decrease diagnostic sensitivity, increasing the rate of false positives (Gagnon et al. 2013; Malek‐Ahmadi et al. 2015). Taken cumulatively, it is critical to use age‐ and education‐corrected normative data when interpreting MoCA performance.

Several studies have provided normative data for the MoCA with consideration of different demographic characteristics, including age, education, race, and ethnicity. However, these studies are limited in that they have included clinic‐based samples (Dautzenberg et al. 2020; Dautzenberg et al. 2021), small sample sizes (Elkana et al. 2020), overrepresentation of older adults (Malek‐Ahmadi et al. 2015) and examined participants residing outside the United States (Aiello et al. 2022; Cervigni et al. 2022; Sun et al. 2023) or those without predominant non‐Hispanic White cultural and ethnic backgrounds (O'Driscoll and Shaikh 2017; Stimmel et al. 2024; Rossetti et al., 2011). As a result, studies have provided a wide range of cutoff scores ranging from 18.5–27 for detection of MCI, with 23 points having been suggested as a revision to the MoCA cutoff score (Carson et al. 2017; Kutash et al. 2023; Ilardi et al. 2023; Islam et al. 2023; Sachs et al. 2022; Stimmel et al. 2024; Thomann et al. 2020).

The lack of consensus regarding appropriate normative data on the MoCA contributes to the continued use of < 26 as the cutoff by clinicians and physicians, resulting in a high rate of referrals to specialists consisting of false positives, which leads to potential mismanagement of resources and increased healthcare costs, ultimately overwhelming medical resources (Dautzenberg et al. 2020; Edmonds et al. 2016; Klekociuk et al. 2014). In addition, the MoCA cutoff scores of 26 and 23 are often used in the selection and categorization of participants with respect to level of cognitive impairment in research studies and clinical trials (Dautzenberg et al. 2020, 2021; Debert et al. 2019; Ilardi et al. 2023) Further, there is a notable gap in MoCA normative data from large population‐based samples with a wide age range (Cecato et al. 2016; Malek‐Ahmadi et al. 2015; Milani et al. 2018) with few studies providing normative data for individuals below the age of 65 years (Bruijnen et al. 2020; Feeney et al. 2016; Larouche et al. 2016; Pike et al. 2017). Some studies, including those with participants as young as 14 use the standard MoCA cutoff score of less than < 26 cutoff to distinguish between healthy controls and cognitively impaired individuals (Cersonsky et al. 2022; Debert et al. 2019; Pike et al. 2017). Establishing demographically corrected norms is therefore essential to ensure accurate participant selection and improve the tool's utility in both clinical and research settings, particularly with young and middle‐aged adults, where literature regarding appropriate normative data is limited.

In this study, we provide age‐ and education‐corrected normative data for the MoCA in cognitively healthy participants of the longest‐running epidemiological cohort study in the United States, the Framingham Heart Study (FHS). While this community‐based sample shares characteristics similar to the original MoCA study population (Nasreddine et al. 2005) in that participants are primarily White and highly educated, the sample size is significantly larger with a broader age range, including middle‐aged individuals, thereby providing updated norms for cognitively healthy individuals for one of the most commonly used cognitive screening instruments. This study seeks to provide (1) scores on the MoCA for cognitively healthy individuals based on age and education as opposed to the utilization of a universally applied cutoff score and (2) normative data for middle‐aged adults, the norms for which are limited in the literature.

## 2 Materials and Methods

### 2.1 Participants

Initiated in 1948, FHS is a population‐based cohort study with regular health exams that occur every 4–8 years (Dawber et al. 1951). The present study included cognitively unimpaired participants (*n = *2637; 91.43% non‐Hispanic White) from Generation 3 (2002–2005; *n = *4095) and a more ethnically diverse Omni 2 (2003–2005) (*n = *410) cohorts who completed the MoCA as part of their third health exam (Splansky et al. 2007). Participants included in the sample had scores of **< **2 on the AD8 Dementia Screening, an 8‐point self‐report questionnaire that assesses functional change (score ≥ 2 is indicative of functional impairment; Galvin et al. 2005). Seventeen participants were excluded as they met criteria for possible cognitive impairment based on dementia adjudication conference, and 694 participants were excluded for an AD8 score of ≥ 2.

Health exams included detailed medical and physical examinations, cognitive screening measures, and laboratory tests. Information about various vascular risk factors, including smoking status, diabetes, treatment for hypertension, systolic BP, total cholesterol, and HDL cholesterol, was obtained. Participants completed a blood draw, from which apolipoproteinE4 (Apoe4) genetic information was determined. The health exams lasted four to 5 hours on average.

### 2.2 Measures

The MoCA is a 30‐item cognitive assessment that takes approximately 10 min to administer (Nasreddine et al. 2005). The MoCA assesses visuospatial/executive functioning, naming, memory, attention, language, abstraction, delayed recall, and orientation. The MoCA was administered by certified personnel who utilized the 1‐point correction for those with ≤ 12 years of education. In addition, as mentioned above, participants were administered the AD8, scores on which were used to determine if participants were showing early cognitive or functional changes.

### 2.3 Standard Protocol Approvals, Registrations, and Patient Consents

All participants provided written informed consent to participate in the health exam, and the study protocol was approved by the Institutional Review Board of Boston University Chobanian & Avedisian School of Medicine.

### 2.4 Statistical Analysis

Descriptive statistics, including mean and standard deviation for continuous variables, and counts and percentages for categorical variables, were generated. Age was divided into 10‐year age bands (< 40, 40–49, 50–59, 60–69, ≥ 70). Participants who graduated from high school and did not graduate from high school were combined into one group due to low sample size. Education levels were further stratified into those with some college education and those with a college degree or higher. Average total MoCA scores were generated by age and education level. Percentile distributions (5th, 10th, 25th, 50th, 75th, 90th, and 95th) of MoCA scores were calculated within each age and education group. Average scores on each cognitive domain on the MoCA were also generated. A two‐way analysis of variance (ANOVA) was used to compare MoCA scores across all age groups and education levels. Tukey's multiple comparison method was used to correct for Type I error rate. Correlations were conducted between AD8 and MoCA scores to examine the relationship between cognitive performance on the MoCA and degree of functional impairment. All statistical analyses were performed using SAS programming software.

## 3 Results

**FIGURE 1:**
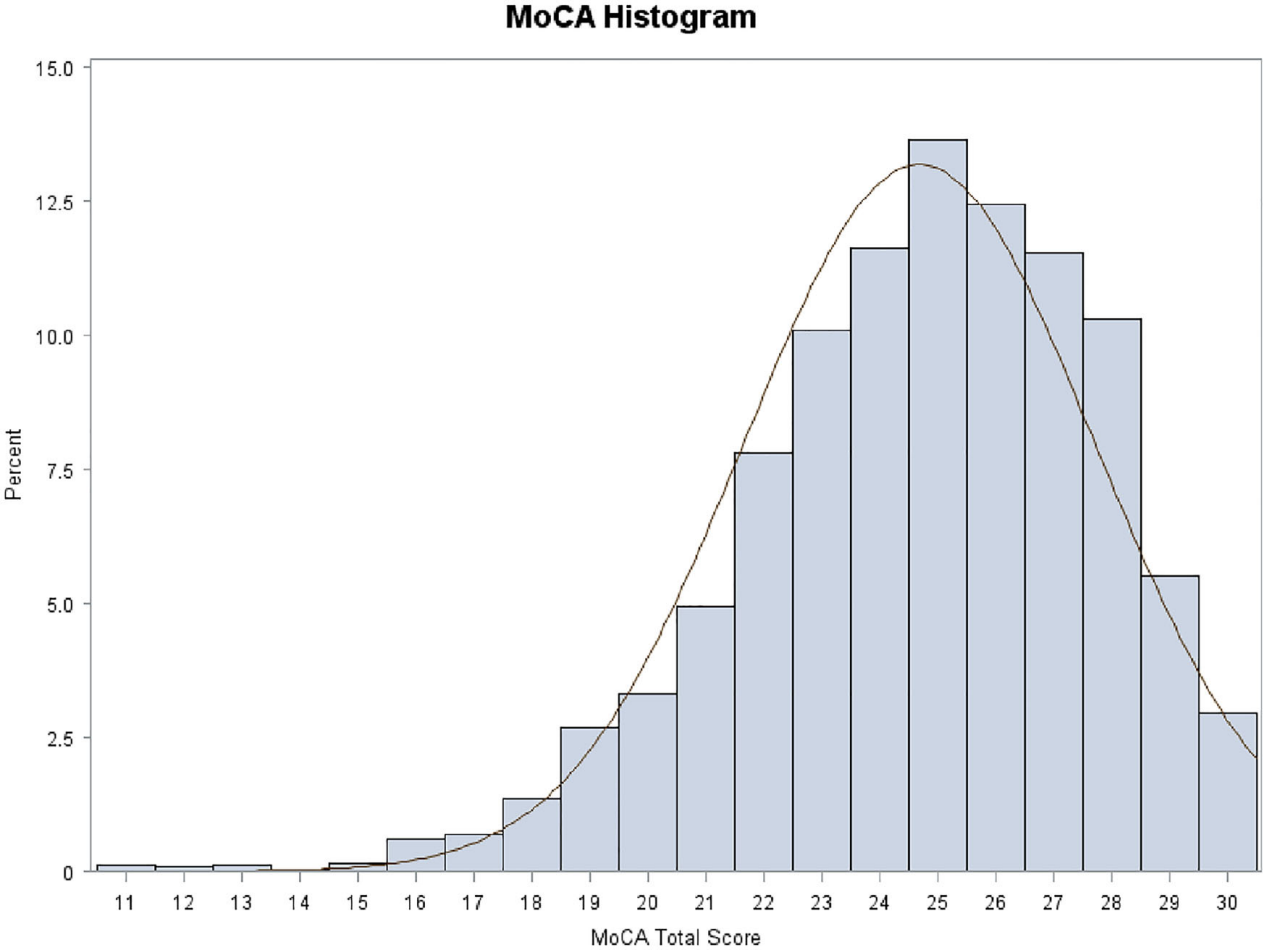
Overall MoCA score distribution. This histogram displays the distribution of Montreal Cognitive Assessment (MoCA) total scores in the Framingham Heart Study sample. The *x*‐axis represents MoCA total scores, ranging from 11 to 30, while the *y*‐axis indicates the percentage of participants achieving each score. The overlaid density curve (brown line) illustrates the estimated probability distribution, showing a right‐skewed distribution with a peak around score of 24.

The sample was 53.05% female, predominantly White, and well‐educated (Mean age = 53.56, SD age = 8.92, age range = 32–83, 63.71% ≥ college educated, 91.43% non‐Hispanic White; Table 1). Percentile distributions of MoCA scores by age and education groups are presented in Table S2. On average, participants scored a total of 24.69 points on the MoCA (SD = 3.03; Figure 1). Specifically, 47 participants had a MoCA score of less than 18; however, participants were largely independent in their functioning (AD8 M = 0.25, SD = 0.43; Table 1). There was a small yet significant negative relationship between participants’ AD8 and MoCA scores (*r = *−0.05, *p = *0.005).

**TABLE 1:** Demographic characteristics.

Demographics		*n*	%
Age			
	Below 40 (min age: 32)	184	6.98
	40–49	666	25.26
	50–59	1096	41.56
	60–69	607	23.02
	70 and above (max age: 83)	84	3.19
Sex			
	Female	1399	53.05
	Male	1238	43.05
Education			
	High school grad or less	242	9.18
	Some college	715	27.11
	College grad or more	1680	63.71
Ethnicity			
	Asian	55	2.09
	Black	45	1.71
	Hispanic	61	2.31
	Multi ethnic	60	2.28
	Native American/ Pacific Islander	2	0.08
	Unknown	1	0.04
	White	2411	91.43

Medical History	*n*	% Yes
Current smoking status	180	6.86
Diabetes	178	6.79
Treated for hypertension	526	20.05
Apoe4 allele present	492	19.63

	M	SD
Systolic blood pressure (mmHg)	118.46	14.29
Total cholesterol (mg/dL)	188.53	36.82
HDL cholesterol (mg/dL)	59.89	18.93

AD8 scores		M	SD
Age			
	Below 40	0.16	0.37
	40–49	0.20	0.40
	50–59	0.25	0.43
	60–69	0.30	0.46
	70 and above	0.44	0.50
Education			
	High school grad or less	0.32	0.47
	Some college	0.30	0.46
	College grad or more	0.22	0.41

*Note*: Systolic blood pressure (Fatani et al. 2021), total cholesterol, and high‐density‐lipoprotein cholesterol values (Rosenson 2025) fall within acceptable ranges. The Washington University Dementia Screening Test: score ≥ 2 = cognitive impairment (Galvin et al. 2005).

Abbreviations: HDL = high density lipoprotein, Apoe4 = apolipoproteinE4; AD8 = The AD8.

There were significant main effects of MoCA scores for both age; *F* (4, 2622) = 15.09, *p* < 0.0001, and education; *F* (2, 2622) = 36.72, *p* < 0.0001, but no interaction effect; *F* (8, 2622) = 1.38, *p = *0.1982. There were significant differences in total MoCA scores between all education groups and for most age groups, with the exception of participants in their 40s not having significantly different MoCA scores than those either below 40 years of age (*p* = 0.808) or those in their 50s (*p* = 0.066). MoCA scores across age and education levels are illustrated in Table 2. As expected, younger participants and those with high education scored higher on the MoCA than those who were older and had low education. Notably, the average MoCA score for individuals below the age of 40 who were college educated was at the original MCI cutoff suggested for the MoCA (M = 26.28, SD = 2.41, *N* = 151; Table 2). Further, MoCA scores for middle‐aged individuals (i.e., individuals in their 40s and 50s) were consistently below the cutoff of 26 points irrespective of level of education (Table 2). Further, the mean score for individuals over the age of 60 was at the recently revised MCI cutoff score of 23 points (Table 2). Similarly, individuals above the age of 70 scored below the revised cutoff score of 23 points irrespective of level of education (Table 2).

**TABLE 2:** Average score on the MoCA stratified by age group and education level.

Education level											
	High school grad or less			Some college			College grad or more			Total by age	
	*n*	M (SD)	95% CI	*n*	M(SD)	95% CI	*n*	M (SD)	95% CI	*n*	M (SD)
Age group			(LL, UL)			(LL, UL)			(LL, UL)		
Below 40	9	23.89 (5.62)	(19.57, 28.21)	24	25.17 (3.78)	(23.57, 26.76)	151	26.28 (2.41)	(25.89, 26.67)	184	26.02 (2.89)
40–49	29	24.07 (4.32)	(22.43, 25.71)	155	23.94 (3.45)	(23.40, 24.50)	482	25.92 (2.43)	(25.70, 26.14)	666	25.37 (2.93)
50–59	114	23.06 (3.13)	(22.48, 23.64)	313	23.84 (2.96)	(23.51, 24.17)	669	25.27 (2.55)	(24.07, 25.46)	1096	24.63 (2.86)
60–69	76	22.29 (3.30)	(21.53, 23.04)	197	23.40 (2.96)	(22.99, 23.82)	334	24.61 (2.85)	(24.30, 24.92)	607	23.93 (3.06)
70 and above	14	20.79 (2.42)	(19.39, 22.19)	26	23.04 (3.86)	(21.48, 24.60)	44	23.05 (3.26)	(22.05, 24.04)	84	22.67 (3.41)
Total by education	242	22.84 (3.48)		715	23.76 (3.15)		1680	25.36 (2.67)		2637	24.69 (3.03)

Abbreviations: CI = confidence interval; LL = lower limit; UL = upper limit.

Average scores for each cognitive domain on the MoCA are listed in Table 3. Notably, participants scored an average of 2.34 out of 5 points (SD = 1.67) on delayed recall across all age groups and education levels (i.e., 71.05% of the sample obtained less than three points on delayed recall). Suboptimal scores on delayed recall persisted even for college‐educated individuals below the age of 40 (M = 3.15; SD = 1.56) (Table S1).

**TABLE 3:** Average MoCA domain score.

Domain	Mean score/total possible (pts.)	Std. dev.
Visuospatial/executive	4.12/5	0.93
Naming	2.92/3	0.30
Digits‐attention	1.86/2	0.39
Letters‐attention	0.91/1	0.28
Serial 7s‐attention	2.69/3	0.69
Repeat sentence‐language	1.57/2	0.61
Fluency‐language	0.79/1	0.41
Abstraction	1.45/3	0.70
Delayed recall	2.35/5	1.67
Orientation	5.92/6	0.31
Total score	24.69/30	3.03

## 4 Discussion

The current study provides normative data by age and education for cognitively healthy participants with demographic characteristics similar to the MoCA's initial validation sample (i.e., highly educated, non‐ Hispanic White individuals), although it differs in that it provides data from a well‐established community‐based cohort with a wider range of ages from 32 to 83 years. This study is unique in that it provides demographically corrected data for cognitively healthy middle‐aged adults, which are found to a limited extent in the literature. Although the MoCA has been used in studies involving midlife adults, these studies are not focused on establishing normative data and instead use predetermined cutoffs to establish MCI prevalence (Jia et al. 2021), examine correlations between MoCA performance and other cognitive measures (Elkana et al. 2022; Quang et al. 2023), or use the MoCA in specific clinical populations (Quang et al. 2023; Raghunath et al. 2021; Haddad et al. 2022). Further, many of these studies are conducted with individuals outside of North America, primarily with individuals from non‐Hispanic White backgrounds (Jia et al. 2021; Quang et al. 2023). Thus, studies call for further research to better understand MoCA's applicability in midlife due to the lack of large, age‐specific normative samples (e.g., Lynch et al. 2022). Results from the present study showed that most cognitively healthy individuals scored below the original cutoff for MCI (i.e., 26 points) and closer to, if not lower than, the recently revised MCI cutoff of 23 points (Carson et al. 2017). Not only do these findings suggest that the original cutoff of 26/30 points produces a high rate of false positives, but they also raises similar concerns for the revised MCI cutoff of 23 points, particularly in those ≥ 60 years of age and with lower education, consistent with previous literature (Ilardi et al. 2023; Ratcliffe et al. 2024; Sachs et al. 2021; Stimmel et al. 2024).

The delayed recall score on the MoCA, an item that is likely to be interpreted as a marker of possible memory problems, was surprisingly low across all age and education levels (M = 2.34, SD = 1.67) in this cognitively healthy sample. This underscores the importance of examining specific items on the MoCA that may be differentially contributing to the overall score. Clinically, the findings underscore the limitations of using a single cutoff across populations: while a cutoff of 26 may be overly stringent for older or less educated groups, it may underestimate impairment risk in younger, more educated individuals. Thus, our results reinforce the use of demographic adjustments as well as percentile‐based data in interpreting MoCA scores. Consistent with other studies (e.g., Malek‐Ahmadi and Nikkhahmanesh 2024), our findings highlight the need to account for various sociocultural and clinical factors when interpreting MoCA scores to inform clinically meaningful decision making, reinforcing its use as a screening measure and not a replacement for a full neuropsychological evaluation.

## 5 Conclusion

Overall, consistent with previous research, our results suggest that without the use of appropriate demographically corrected normative data and interpretive considerations, performance on the MoCA may result in false positives in clinical practice and incorrect classification in clinical research studies (Chun et al. 2021; Dautzenberg et al. 2021; Edmonds et al. 2016; Klekociuk et al. 2014). In addition to using appropriate normative data for the MoCA, growing research emphasizes the need for trained professionals to interpret MoCA scores in conjunction with a clinical interview and medical history review to improve diagnostic accuracy (Reimers 2019; Creavin et al. 2021; Maki et al. 2021). To this effect, 10 of 47 participants who scored less than 18 on the MoCA were deemed to be cognitively unimpaired based on follow‐up neuropsychological testing and further clinical review. The remaining 37 individuals with a MoCA score of < 18 are being actively recruited for further neuropsychological testing, which serves as a limitation for the present study, although the average AD8 score of these individuals was 0.16 (range: 0–1), making cognitive impairment less likely. A potential explanation for MoCA scores being incongruent with participants' functional and cognitive status is that the MoCA in the current study was administered at variable times as part of a 4‐to‐5‐hour health exam, which could result in variables such as fatigue, disinterest, or fasting for blood tests impacting performance.

Limitations of the current study include a lack of diverse representation, limiting the generalizability of findings to highly educated and non‐Hispanic White individuals. Given the relatively small proportion of participants who were non‐White in this sample, MoCA scores were not stratified based on race/ethnicity. In addition, the sample size of individuals ≤ 40 and ≥ 70 years is small, especially when categorized by education, potentially limiting its utility. Future research involves scoring of the MoCA using item response theory to account for item difficulty and participants' ability (Luo et al. 2020; Benitez et al. 2015; Mungas and Reed 2000) as opposed to the current scoring of the MoCA that uses classical test theory (Hambleton and Jones 1993), which is less psychometrically appropriate for longitudinal tracking purposes. Providing cutoff scores for different clinical groups, including MCI and dementia, is also a direction for future research. In conclusion, while the MoCA remains a widely used cognitive screening tool in both research and clinical settings, findings from the current study highlight the need for critical evaluation of currently used cutoff scores and use of appropriate demographically corrected norms, especially for middle‐aged adults, for whom norms are limited in the literature.

## Author Contributions

**Emma Muller**: conceptualization, curation, visualization, writing‐ original draft, writing – review and editing. **Calvin Guan**: curation, analysis, visualization, writing – review and editing. **Katherine A. Gifford**: writing – review and editing. **Preeti Sunderaraman**: writing – review & editing. **Sherral Devine**: writing – review and editing. **Yulin Liu**: analysis, writing – review and editing. **Phillip H. Hwang**: writing – review and editing. **Ashita S. Gurnani**: conceptualization, funding, investigation, methods, supervision, writing – original draft, writing – review and editing.

## Ethics Statement

This study was approved by the Institutional Review Board of Boston University Chobanian & Avedisian School of Medicine.

## Consent

Written informed consent was obtained from all participants in accordance with the study protocol.

## Conflicts of Interest

The authors declare no conflicts of interest.

## Supporting information



Supplementary Table 1. MoCA Domain/Item Scores by Age Group and Education Level

Supplementary Table 2. Percentile Distributions of MoCA Scores by Age & Education

## Data Availability

The data used in this study were obtained from the Framingham Heart Study and are publicly available.
