# Supplementary material for: The Montreal Cognitive Assessment at the Framingham Heart Study: A Re‐Examination of the Norms
Source: Brain Behav. 2026 May 11;16(5):e71487. doi: 10.1002/brb3.71487 (PMC13159545; doi:10.1002/brb3.71487)
Supplement: Supplementary file 1 — Supplementary Table 1. MoCA Domain/Item Scores by Age Group and Education Level [file BRB3-16-e71487-s001.docx]

Supplementary Table 1. MoCA Domain/Item Scores by Age Group and Education Level

| Age Group | Education Level |  | Domain/Item *M (SD)* | | | | | | | | | |
| --- | --- | --- | --- | --- | --- | --- | --- | --- | --- | --- | --- | --- |
|  |  | *n* | Visuospatial/ Executive  (5 points) | Naming  (3 points) | Digits- Attention  (2 points) | Letters- Attention (1 point) | Serial 7s- Attention  (3 points) | Repeat Sentence- Language  (2 point) | Fluency- Language  (1 point) | Abstraction  (3 points) | Delayed Recall  (5 points) | Orientation  (6 points) |
| Below 40 | High school grad or less | 9 | 4.33 (0.87) | 2.89 (0.33) | 1.78 (0.67) | 0.78 (0.44) | 2.38 (1.06) | 1.44 (0.73) | 0.67 (0.50) | 1.11 (0.78) | 2.11 (1.45) | 5.67 (1.00) |
| Below 40 | Some college | 24 | 4.38 (0.82) | 2.96 (0.20) | 1.87 (0.34) | 0.92 (0.28) | 2.46 (1.02) | 1.46 (0.78) | 0.75 (0.44) | 1.46 (0.66) | 2.79 (1.72) | 5.96 (0.20) |
| Below 40 | College grad or more | 151 | 4.46 (0.77) | 2.99 (0.12) | 1.92 (0.30) | 0.93 (0.25) | 2.78 (0.60) | 1.70 (0.56) | 0.87 (0.34 | 1.52 (0.65) | 3.15 (1.56) | 5.95 (0.24) |
| 40 - 49 | High school grad or less | 29 | 3.83 (1.10) | 2.90 (0.31) | 1.76 (0.64) | 0.90 (0.31) | 2.45 (1.02) | 1.28 (0.80) | 0.69 (0.47) | 1.28 (0.65) | 2.14 (1.83) | 5.97 (0.19) |
| 40 - 49 | Some college | 155 | 3.87 (0.97) | 2.93 (0.26) | 1.85 (0.39) | 0.89 (0.31) | 2.50 (0.87) | 1.45 (0.68) | 0.73 (0.44) | 1.26 (0.74) | 2.39 (1.72) | 5.92 (0.30) |
| 40 - 49 | College grad or more | 482 | 4.38 (0.84) | 2.95 (0.26) | 1.88 (0.34) | 0.93 (0.25) | 2.82 (0.49) | 1.66 (0.54) | 0.84 (0.37) | 1.56 (0.63) | 2.98 (1.55) | 5.92 (0.37) |
| 50 - 59 | High school grad or less | 114 | 3.65 (1.11) | 2.96 (0.21) | 1.72 (0.51) | 0.91 (0.28) | 2.35 (0.95) | 1.35 (0.70) | 0.66 (0.48) | 1.03 (0.81) | 1.59 (1.41) | 5.88 (0.36) |
| 50 - 59 | Some college | 313 | 3.98 (0.91) | 2.93 (0.26) | 1.81 (0.45) | 0.90 (0.30) | 2.58 (0.77) | 1.47 (0.61) | 0.74 (0.44) | 1.28 (0.76) | 2.03 (1.61) | 5.92 (0.29) |
| 50 - 59 | College grad or more | 669 | 4.24 (0.82) | 2.92 (0.29) | 1.88 (0.33) | 0.94 (0.23) | 2.79 (0.54) | 1.66 (0.53) | 0.83 (0.38) | 1.57 (0.63) | 2.51 (1.61) | 5.92 (0.28) |
| 60 - 69 | High school grad or less | 76 | 3.57 (1.02) | 2.79 (0.44) | 1.76 (0.49) | 0.80 (0.40) | 2.35 (0.85) | 1.17 (0.77) | 0.67 (0.48) | 1.09 (0.86) | 1.37 (1.54) | 5.93 (0.25) |
| 60 - 69 | Some college | 197 | 3.91 (0.99) | 2.88 (0.33) | 1.86 (0.35) | 0.86 (0.35) | 2.50 (0.88) | 1.55 (0.58) | 0.72 (0.45) | 1.37 (0.77) | 1.72 (1.55) | 5.89 (0.36) |
| 60 - 69 | College grad or more | 334 | 4.15 (0.88) | 2.90 (0.33) | 1.88 (0.34) | 0.90 (0.30) | 2.77 (0.58) | 1.56 (0.65) | 0.81 (0.40) | 1.59 (0.63) | 2.11 (1.62) | 5.94 (0.23) |
| 70 and above | High school grad or less | 14 | 3.57 (0.85) | 2.64 (0.63) | 1.57 (0.51) | 0.86 (0.36) | 2.21 (1.12) | 1.14 (0.54) | 0.36 (0.50) | 1.07 (0.83) | 0.57 (0.94) | 5.86 (0.36) |
| 70 and above | Some college | 26 | 3.38 (1.33) | 2.85 (0.37) | 1.85 (0.37) | 0.77 (0.43) | 2.77 (0.59) | 1.46 (0.71) | 0.81 (0.40) | 1.31 (0.84) | 1.88 (1.75) | 5.77 (0.43) |
| 70 and above | College grad or more | 44 | 3.70 (1.13) | 2.84 (0.53) | 1.86 (0.46) | 0.89 (0.32) | 2.68 (0.64) | 1.52 (0.70) | 0.70 (0.46) | 1.57 (0.63) | 1.34 (1.54) | 5.93 (0.26) |
