# Supplementary material for: The Montreal Cognitive Assessment at the Framingham Heart Study: A Re‐Examination of the Norms
Source: Brain Behav. 2026 May 11;16(5):e71487. doi: 10.1002/brb3.71487 (PMC13159545; doi:10.1002/brb3.71487)
Supplement: Supplementary file 2 — Supplementary Table 2. Percentile Distributions of MoCA Scores by Age & Education [file BRB3-16-e71487-s002.docx]

Supplementary Table 2. Percentile Distributions of MoCA Scores by Age & Education

| Age Group | 5th% | 10th% | 25th% | Median (50th%) | 75th% | 90th% | 95th% | *N* |
| --- | --- | --- | --- | --- | --- | --- | --- | --- |
| below 40 | 20 | 22 | 24 | 27 | 28 | 29 | 30 | 184 |
| 40 - 49 | 20 | 22 | 24 | 26 | 27 | 29 | 29 | 666 |
| 50 - 59 | 19 | 21 | 23 | 25 | 27 | 28 | 29 | 1096 |
| 60 - 69 | 18 | 20 | 22 | 24 | 26 | 28 | 29 | 607 |
| 70 and above | 16 | 18 | 21 | 23 | 25 | 27 | 28 | 84 |
| Education Group | | |  |  |  |  |  |  |
| High school grad or less | 17 | 18 | 21 | 23 | 25 | 27 | 29 | 242 |
| Some college | 18 | 20 | 22 | 24 | 26 | 28 | 28 | 715 |
| College grad or more | 21 | 22 | 24 | 26 | 27 | 29 | 29 | 1680 |
